# Primary Aldosteronism and Long‐Term Cardiovascular Complications: Comparison of Medical Versus Surgical Treatment

**DOI:** 10.1111/jch.70128

**Published:** 2025-08-22

**Authors:** Sofia Benameur, Julien Bertolino, Laura Bonnaud, Ngoc Anh Thu Nguyen, Barbara Leclercq, François Silhol, Frederic Castinetti, Frederic Sebag, Bernard Vaisse, Gabrielle Sarlon‐Bartoli

**Affiliations:** ^1^ Vascular Medicine and Arterial Hypertension Department La Timone Hospital Marseille France; ^2^ Medical Evaluation Service AP‐HM, CIC‐CPCET Marseille France; ^3^ Endocrinology Department La Conception Hospital Marseille France; ^4^ General Endocrine and Metabolic Surgery Department La Conception Hospital Marseille France

**Keywords:** cardiovascular, primary aldosteronism, secondary high blood pressure (hbp)

## Abstract

The study aims to evaluate the long‐term incidence of cardiovascular events (CVE) and compare the effectiveness of medical and surgical interventions using a combined cardiovascular endpoint in individuals diagnosed with primary aldosteronism (PA). The authors carried out a multicentric, retrospective study in Marseille on a total of 106 inpatients divided into two samples with biologically proven primary aldosteronism, of whom 55 underwent surgical treatment and 51 received medical therapy between January 2014 and December 2022. The mean age of the sample was 53 years. Over a 54‐month follow‐up period, five patients in the medical group (10.64%) and three in the surgical group (5.45%) experienced a CVE (*p *= 0.46). Although the difference was not statistically significant, the surgical group had more cardiovascular morbidity at baseline. At the end of the follow‐up, the surgical group demonstrated a significant reduction in blood pressure (BP) (mean 126/74 mmHg) compared to the medical group (mean 136/81 mmHg) (*p *= 0.02), with a significantly lower number of antihypertensive medications (1.23 ± 1.5 vs. 2.83 ± 1.8, *p* < 0.01). Additionally, the surgical group had a significantly higher serum potassium level at the end of follow‐up despite similar potassium supplementation. The long‐term incidence of CVE in PA did not significantly differ between medical and surgical treatment. However, there appears to be a trend toward reduced CVE over the long term in surgically treated patients who had excess cardiovascular morbidity at baseline. In addition, surgical treatment significantly improved BP control, with patients requiring fewer and demonstrating better serum potassium regulation.

## 1 Introduction

Primary aldosteronism (PA), once thought to be a rare condition, is now recognized as potentially the most common curable cause of arterial hypertension. It accounts for 5%–10% of all hypertension cases and even more in the populations of specialist centers or cohorts of resistant hypertension (20%). Contrary to notions of it being a benign form of hypertension, PA is associated with a higher risk of cardiovascular complications, including left ventricular hypertrophy, myocardial infarction, atrial fibrillation, stroke, microalbuminuria, osteoporosis, and metabolic syndrome.

Previous studies involving larger patient populations have confirmed the pathogenic role of aldosterone in the cardiovascular system, highlighting the importance of early diagnosis of this condition. The results demonstrate that cardiovascular complications occur more frequently in patients with PA compared to those with essential hypertension, despite comparable cardiovascular risk profiles. This difference in cardiovascular event rates can be reversed by suppressing the effects of excess aldosterone, either through adrenalectomy or treatment with mineralocorticoid receptor antagonists (MRAs).

Although adrenalectomy is currently considered the standard treatment for patients with unilateral PA, some may have residual hypertension and still require antihypertensive medication after surgery. Resistance to medical treatment can occur in some patients.

Previously published studies have compared the relative efficacy of surgical versus medical therapy for PA but offer contradictory findings. For instance, one study showed that surgery for PA led to better control of hypertension and hypokalemia compared to medication [1]. Another recent paper found that patients with unilateral PA who underwent surgery experienced a significant reduction in left ventricular mass after 2.5 years of follow‐up, compared to those with bilateral PA who were treated medically [2]. Additionally, surgical interventions prevent common side effects of MRAs and lessen the limitations associated with lifelong pharmaceutical treatment. Although available evidence suggests that surgery may be more effective than medical therapy for managing blood pressure (BP) and serum potassium levels, there are limited data supporting its capacity to reduce cardiovascular complications.

The primary aim of this paper is to assess the long‐term incidence of cardiovascular events (CVE) in patients with primary hyperaldosteronism by comparing the effectiveness of medical and surgical treatments.

## 2 Materials and Methods

### 2.1 Population

A multicentric retrospective descriptive analysis was conducted at the departments of endocrine surgery at Hôpital de la Conception and vascular medicine at Hôpital de la Timone in Marseille. The study enrolled patients with biologically confirmed primary aldosteronism (PA) between January 2014 and December 2022. We determined biological evidence of PA with two renin/aldosterone ratios (ARR) >64 pmol/mIU (3.6 ng/ng), measured under standardized conditions on two separate occasions, and by plasma aldosterone concentration >550 pmol/L (20 ng/dL). For ARR calculation, active renin concentrations <5 mIU/L (3 ng/L) were set to 5 mIU/L to prevent overestimation of the ARR when active renin was undetectable or present at very low concentrations.

Records were collected from medical data recording systems (PMSI) at both centers using the following keywords: primary hyperaldosteronism, adrenal adenoma, adrenal nodule, and bilateral adrenal hyperplasia. Selected files were then reviewed to ensure conformity with inclusion and exclusion criteria.

Criteria for inclusion were as follows: patients had to be under the care of one of the two centers and have confirmed primary aldosteronism (PA), as determined by the measurement of plasma renin and plasma aldosterone concentration with at least 2 ARR >64 pmol/mIU under neutral treatment. All patients underwent a computed tomography (CT) scan of the adrenal glands. Adrenal vein sampling (AVS) was performed if necessary to determine suitability for surgery. The AVS is the gold standard exam for distinguishing unilateral or bilateral aldosterone secretion in PA. AVS validity is determined by a selectivity index higher than 2 and is calculated based on the cortisol ratio in the adrenal veins on the cortisol of the vena cave. If the selectivity index is below 2, the results of the AVS cannot be interpreted. The lateralization was confirmed when the index was above 4. The lateralization index corresponds to the ratio of aldosterone to cortisol between the dominant and other adrenal veins. Only patients with lateralized secretion underwent surgery. Subsequently, patients were categorized into the surgical group if they had undergone adrenalectomy, or into the medical group if they had not, but were receiving at least one specific treatment for PA.

Exclusion criteria included non‐confirmation of PA on biological assays, no aldosterone‐blocking therapy in the medical group, PA of genetic origin, diagnoses made before 2014 (due to unavailable or incomplete computerized medical records), and records with insufficient data.

### 2.2 Data Collection and Phone Calls

The following information was gathered retrospectively from the patients’ computerized medical records (AXIGATE software): demographic data, family history, medical history, cardiovascular risk factors, CVE, imaging data, biology, blood pressure values, and treatments. We set the date of diagnosis of PA as the inclusion date.

In the follow‐up phase of the study, participants were contacted by phone to obtain their consent for the collection of their medical data. In May 2023, they were also asked to answer a mandatory questionnaire regarding their history, their last biological analysis, and the occurrence of any CVE since the last consultation. The collected data allowed us to confirm the information obtained from the medical records and to complete the missing data. Furthermore, patients were invited to perform self‐measurement at home using the following rule: average blood pressure over 3 days, with three measurements taken in the morning and three in the evening. A minimum 6‐month follow‐up was required for inclusion.

### 2.3 End Points

The primary endpoint was to assess the long‐term incidence of cardiovascular events (CVE) using a cardiovascular composite score in patients with PA by comparing medical and surgical treatment. The cardiovascular composite score included cardiovascular death, stroke, myocardial infarction, stable angina, atrial fibrillation, hospitalization for heart failure, and aortic dissection.

Secondary aims were to compare the two groups’ blood pressure values, medication usage, and serum potassium levels.

### 2.4 Ethics

The study was documented using the AP‐HM Health Data Access Portal, which allows data collection and anonymization. All methods were used in compliance with approved guidelines. Oral consent was obtained over the phone for all patients.

### 2.5 Statistics

Descriptive and comparative analyses were carried out using IBM SPSS Statistics version 20.0 (IBM, Armonk, NY, USA). Quantitative variables were described using the mean (±SD) and median [IQR]. Qualitative variables were described by number and percentage (%). For univariate analysis, quantitative variables were compared using either a Student's or Mann‐Whitney test. Qualitative variables were compared using a Chi^2^ (*X* ^2^) or Fisher test when appropriate. A *p* value < 0.05 was considered significant.

## 3 Results

### 3.1 Patients’ Characteristics

We pulled 792 records from the PMSI database between 2014 and 2022. The diagnosis of PA was confirmed in 106 patients. Fifty‐five patients were treated surgically. A total of 51 patients were treated medically. In the medical group, 11 patients had an AVS who confirmed unilateral PA with adenoma. But these 11 patients refused surgery. Six patients had an adenoma with a suspected unilateral secretion but do not performed an AVS for confirmation. Thirty five patients did not have adenoma, but only 12 underwent an AVS for confirmed bilateral secretion.

The clinical and biological data for PA patients treated medically and surgically are summarized in Table 1.

**TABLE 1:** Population characteristics.

	Medical treatment	Surgical treatment	*p* value
Gender			
Male	2 (18.2%)	27 (49.1%)	0.057
Female	9 (81.8%)	28 (50.9%)	
Age	58.3 ± 7.7	53.7 ± 11.5	0.054
BMI (kg/m^2^)	25.2 ± 3.7	27.8 ± 4.7	0.095
Heart disease			
CAD	0 (0%)	8 (17%)	0.07
Dilated heart disease	1 (10%)	0 (0%)	
PAD	0 (0%)	2 (4.5%)	0.23
Stroke			
Ischemic	0 (0%)	3 (6.8%)	0.9
Hemorrhagic	0 (0%)	0 (0%)	
Serum potassium (mmol/L)	3.6 ± 0.5	3.3 ± 0.4	0.087
Serum creatinine (µmol/L)	78 ± 26.8	78.05 ± 26.8	0.19
Aldostérone (pmol/L)	765.64 ± 423.2	1112.49 ± 982.1	0.067
Renin (mUI/L)	3.3 ± 1.4	5.3 ± 4.7	0.17
ARR	150.7 ± 84	197.5 ± 98	0.34

Abbreviations: ARR, Aldosteron Renin Ratio; BMI, body mass index; CAD, coronary artery disease; CPAP, continuous positive airway pressure; CVE, cardiovascular event; GFR, glomerular filtration rate; LDL, low density lipoprotein; PAD, peripheral arterial disease.

The demographic data revealed no significant differences between the two groups. The average age of the patients was 53 years (±11 years), with a discrete female predominance (54% in the medical group vs. 50.9% in the surgical group) and a marginal difference in overweight status (BMI 27.5 vs. 28.9 kg/m^2^, respectively in the medical and surgical groups). The majority of patients were Caucasian.

Concerning cardiovascular risk factors, the two groups exhibited a mean Grade 1 hypertension with no significant difference between them (systolic BP: respectively 153 vs. 147 ± 20 mmHg, and diastolic: 91 vs. 87 ± 14 mmHg, respectively) (Table 2). Similarly, there was no significant difference between the two groups in terms of low‐density lipoprotein (LDL) cholesterol levels (*p *= 0.10), active or former smoking habits (*p *= 0.33 and 0.72), and the presence of diabetes (*p *= 0.78).

**TABLE 2:** Comparison of blood pressure, number of antihypertensive classes, and serum potassium level between medical and surgical treatment.

		Medical treatment *N1 = 51*	Surgical treatment *N2 = 55*	*p* value
At inclusion				
Office BP	SBP	153.3 ± 19.2	147.13 ± 20.1	0.18
*N1 = 31*	DBP	91.9 ± 13.5	87.26 ± 14	0.15
*N2 = 47*	HR	85 ± 20.9	74.63 ± 13.4	0.07
24 ABPM	SBP	144.8 ± 20.5	139.63 ± 18.8	0.71
*N1 = 26*	DBP	90.1 ± 10.7	87.13 ± 14.5	0.62
*N2 = 8*	HR	74.3 ± 10	71.38 ± 11.3	0.51
HBPM	SBP	152.9 ± 20.1	155.00 ± 35.4	1
*N1 = 8*	DBP	94.3 ± 16.8	95.00 ± 0	0.46
*N2 = 2*	HR	66.3 ± 6.1	63.50 ± 2.1	1
No. of antihypertensive classes		2.1 ± 1.2	2.31 ± 1.3	0.33
Serum potassium		3.6 ± 0.5	3.36 ± 0.4	0.02
Potassium dose in mg		2900 ± 1674	3147 ± 2363	0.7
At the end of follow‐up				
HBPM	SBP	136.5 ± 14.3	126.82 ± 19.2	0.02
*N1 = 35*	DBP	81.1 ± 10.2	74.42 ± 20.7	0.11
*N2 = 39*	HR	48 ± 1	76.80 ± 18.3	0.33
24 h ABPM	SBP	129.4 ± 19	123.50 ± 10.6	0.41
*N1 = 15*	DBP	85.3 ± 17.3	80.00 ±1.4	0.37
*N2 = 2*	HR	76 ± 9.5	78.50 ± 7.8	0.67
Office BP	SBP	137.7 ± 12.26	148 ± 0	0.47
*N1 = 16*	DBP	82.6 ± 11.6	104 ± 0	0.18
*N2 = 1*	HR	76.6 ± 12.34	93 ± 0	0.21
No. of antihypertensive classes		2.8 ± 1.8	1.2 ± 1.5	<0.005
Serum potassium		4.1 ± 0.54	4.4 ± 0.40	0.01
Potassium dose in mg		933 ± 305	1800 ± 0	0.02

Abbreviations: ABPM, ambulatory blood pressure monitoring; BP, blood pressure; DBP, diastolic blood pressure; HBPM, home blood pressure monitoring; HR, heart rate; SBP, systolic blood pressure.

Conversely, there was a significant difference in the history of stented coronary artery disease between the two groups, with eight patients in the surgical group and two in the medical group (*p* = 0.05). The duration of hypertension at the time of PA diagnosis averaged 17 years (±4) for patients with a history of coronary disease and 5 years for patients without. Patients with a history of coronary disease had a longer evolution of hypertension before PA diagnosis. However, we did not have the date of the coronary events.

Both groups had a similar history of ischemic strokes, but the medical group had a higher number of hemorrhagic strokes, due to significant neurological sequelae that prevented surgery. This could suggest a longer duration of hypertension in the medical group. However, we found that it was similar in both groups, with an average evolution of 8 years (*p *= 0.15). There was no difference in the first‐degree family history of cardiovascular disease.

At diagnosis, the surgical group showed a higher secretion of aldosterone compared to the medical group in hormonal tests (Aldosterone: 1112 pmol/L and ARR 197 in the surgical group; Aldosterone: 710 pmol/L and ARR 127 in the medical group, *p* = 0.007 and *p* = 0.02).

### 3.2 Comparison of Cardiovascular Events (CVE) Between the Two Groups

**FIGURE 1:**
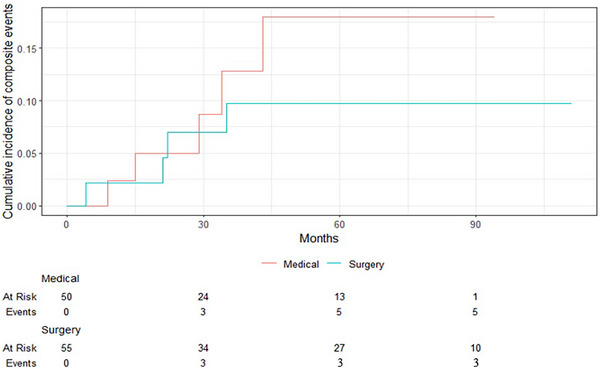
Kaplan–Meier plot showing cardiovascular incidence (myocardial infarction, stroke, revascularization procedures, and sustained arrhythmias) during follow‐up in patients with primary hyperaldosteronism comparing medical and surgical treatment.

The mean duration of follow‐up was 54 months, that is, 4.5 years (±2). In the medical group, five patients, and in the surgical group, three patients achieved the primary endpoint (*p *= 0.46) (Table 3). The difference was not statistically significant. Observed events in the medical group were: myocardial infarction in two patients, stable coronary artery disease with stenting, ischemic stroke and hemorrhagic stroke. In the surgical group, there were two ischemic strokes, and one stented stable coronary artery disease. During the follow‐ups, no patient died or was hospitalized for heart failure. None developed atrial fibrillation. However, there appears to be a trend toward an increased risk of CVE in the medical group compared to the surgical group over the long term (Figure 1).

**TABLE 3:** Comparison of cardiovascular events.

Cardiovascular events	Medical treatment	Surgical treatment	*p* value
Myocardial infarction	2 (4%)	0 (0%)	0.22
Coronary artery disease	1 (2%)	1 (1.8%)	1
Stroke			
Ischemic	1 (2%)	2 (3.6%)	0.79
Hemorrhagic	1 (2%)	0 (0%)	
Deaths	0	0	
Heart failure	0	0	
Atrial fibrillation	0	0	
Number of events	5 (10.6%)	3 (5.4%)	0.46

### 3.3 Comparison of Blood Pressure and Biological Monitoring

**FIGURE 2:**
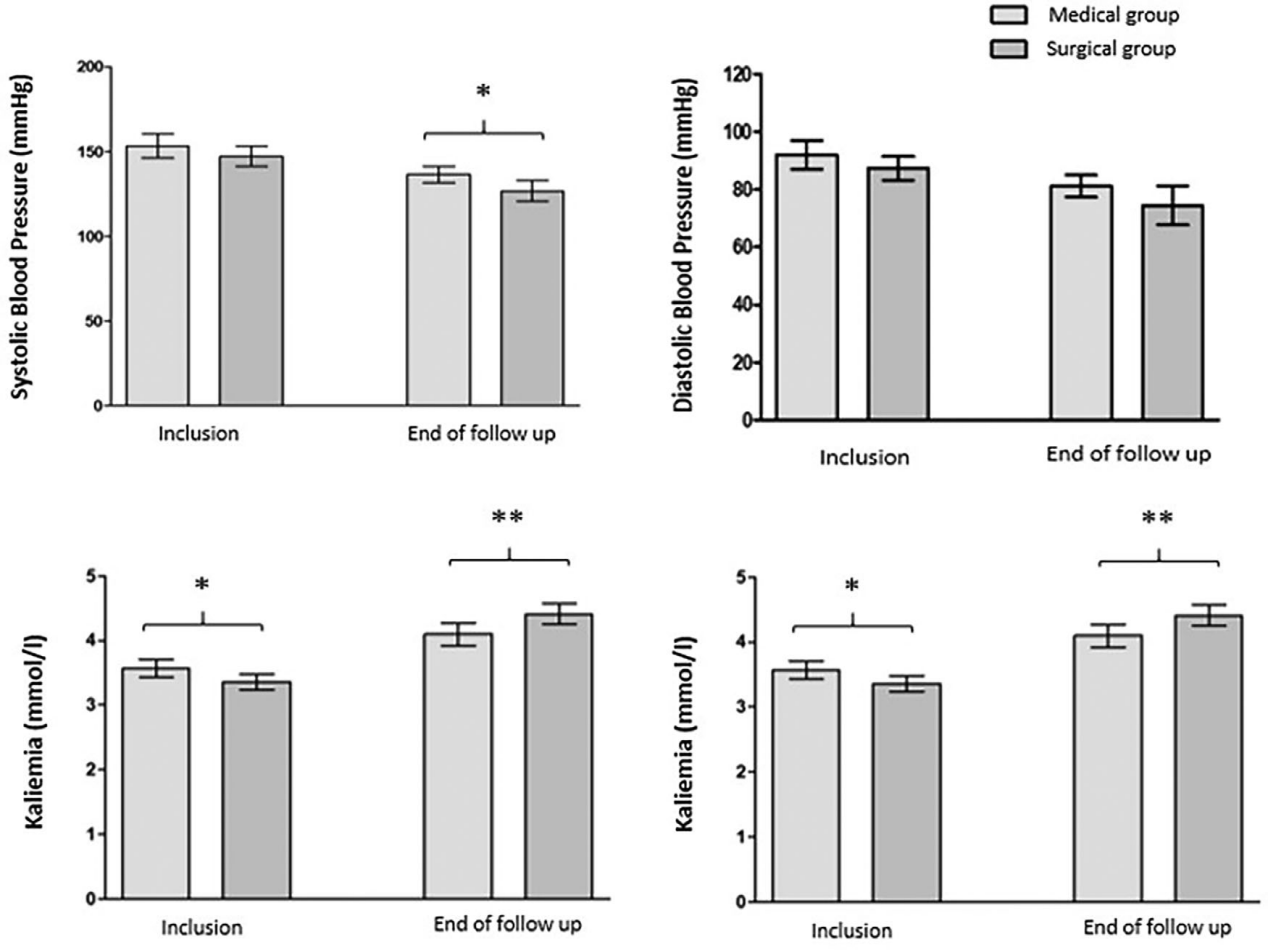
Histograms comparing the blood pressure, number of antihypertensive classes, and serum potassium level, at baseline and at the end of follow‐up for PA patients who had received medical or surgical therapy. * *p* < 0.05 ** *p* < 0.01 *** *p* < 0.001.

Baseline BP was similar in both groups, regardless of the measurement method used, whether it was clinical measurement during a consultation, Home Blood Pressure Monitoring (HBPM) or Ambulatory Blood Pressure Monitoring (ABPM). At inclusion, BP was recorded as 153/91 mmHg in the medical group and 147/87 mmHg in the surgical group (*p *= 0.18 and 0.15). The results show no significant differences in the number of antihypertensive classes use (2.06 ± 1.24 in the medical group vs. 2.31 ± 1.35 in the surgical group). It is important to underline that we chose the office blood pressure to compare the two groups at inclusion because it was the method for which we had the most data (Table 2; Figure 2).

At the end of the follow‐up, we chose HBPM to compare the two groups for the same reason. There was a significant reduction in BP in the surgical group (mean 126/74 mmHg) compared to the medical group (mean 136/81 mmHg) (*p *= 0.02), with a significantly lower number of antihypertensive medications in the surgical group (1.23 ± 1.5 vs. 2.83 ± 1.8, *p *= 0.00005) (Table 2; Figure 2). The medical group was mainly treated with anti‐aldosterone treatment. Details of the different drug classes used in the two groups at inclusion and end of follow‐up are detailed in the Appendix.

The serum potassium levels were significantly lower in the surgical group compared to the medical group (3.36 vs. 3.57 mmol/L, *p *= 0.02), despite similar potassium supplementation (*p* = 0.7). Throughout the follow‐up, plasma potassium concentrations in patients with PA increased significantly from baseline levels, resulting in a notably higher serum potassium level in the surgical group than in the medical group (*p *= 0.01) at the end of follow‐up. It is worth noting that there was no potassium supplementation in the surgical group, with only one patient receiving potassium supplementation in the surgical group as opposed to four in the medical group (Table 2; Figure 2).

### 3.4 Comparison of the Subgroup of Patients With Confirmed Unilateral Hyperaldosteronism by Adrenal Vein Sampling and Treated Medically Versus Those Treated Surgically

We studied a subgroup of patients with unilateral PA confirmed by AVS who refused surgery and were treated medically. These patients were compared with the surgically treated group (Table 4). There was no significant difference between the two groups. At baseline, the plasma aldosterone level was 765 pmol/L in the medical group versus 1112 pmol/L in the surgical group (*p* = 0.067). One CVE occurred in the medical group. There was a trend toward improved blood pressure in the surgical group (126/74 vs. 138/80 in the medical group; *p* = 0.15) despite a lower number of antihypertensive medications (1.2 in the surgical group vs. 2.1 in the medical group; *p* = 0.084).

**TABLE 4:** Comparison of the subgroup of patients with confirmed unilateral hyperaldosteronism by adrenal vein sampling and treated medically versus those treated surgically.

	**Medical treatment** *N1 = 11*	**Surgical treatment** *N = 55*	*p* value
Gender			
Male	2 (18.2%)	27 (49.1%)	0.057
Female	9 (81.8%)	28 (50.9%)	
Age	58.3 ± 7.7	53.7 ± 11.5	0.054
BMI (kg/m^2^)	25.2 ± 3.7	27.8 ± 4.7	0.095
Heart disease			
CAD	0 (0%)	8 (17%)	0.07
Dilated heart disease	1 (10%)	0 (0%)	
PAD			
Stroke	0 (0%)	2 (4.5%)	0.23
Ischemic	0 (0%)	3 (6.8%)	0.9
Hemorrhagic	0 (0%)	0 (0%)	
Serum potassium (mmol/L)	3.6 ± 0.5	3.3 ± 0.4	0.087
Serum creatinine (µmol/L)	78 ± 26.8	78.05 ± 26.8	0.19
Aldostérone (pmol/L)	765.64 ± 423.2	1112.49 ± 982.1	0.067
Renin (mUI/L)	3.3 ± 1.4	5.3 ± 4.7	0.17
ARR	150.7 ± 84	197.5 ± 98	0.34
SBP at inclusion (mmHg)	139.8 ± 13.8	147.1 ± 20.1	0.18
DBP at inclusion (mmHg)	89.9 ±15.7	87.3 ± 14	0.8
No. of antihypertensive classes at inclusion	1.36 ± 0.8	2.31 ±1.3	0.092
SBP at last visit (mmHg)	138.8 ± 20.3	126.8 ± 19.2	0.15
DBP at last visit (mmHg)	80.6 ± 6.8	74.4 ± 20.7	0.14
No. of antihypertensive classes at last visit	2.1 ± 1.3	1.2 ± 1.5	0.084
Number of CVE events	1 (9%)	3 (5.4%)	0.53

Abbreviations: ARR, Aldosteron Renin Ratio; BMI, body mass index; CAD, coronary artery disease; CVE, cardiovascular event; DBP, diastolic blood pressure; PAD, peripheral arterial disease; SBP, systolic blood pressure.

## 4 Discussion

Our study aimed to consider the long‐term incidence of CVE in patients with PA based on their medical or surgical treatment. In our analysis of 106 patients with PA, we observed no significant difference in the occurrence of long‐term cardiovascular complications between medically or surgically treated patients. However, the Kaplan–Meier curves suggest a potential increase in CVE in the medical group over the long term. The extent to which this observation has wider relevance remains unclear and has to be tested on more patients and with longer follow‐up periods. Moreover, surgical patients had a greater history of coronary heart disease at inclusion, indicating a potentially higher cardiovascular risk, despite both groups having similar durations of uncontrolled hypertension, averaging 8 years.

Our findings align with the study conducted by Mulatero et al. [3], which compared the different subtypes of PA, including unilateral PA and bilateral adrenal hyperplasia. Their study revealed that patients with unilateral PA had higher levels of CVE prior to adrenalectomy in comparison to those with bilateral adrenal hyperplasia. The findings also indicate that patients with unilateral secretion exhibit a poorer prognosis than bilateral adrenal hyperplasia, which is probably due to higher aldosterone secretion. This is consistent with our database, which found a higher secretion of aldosterone in the surgical group compared to the medical group at diagnosis (Aldosterone: 1112 pmol/L and ARR 197 in the surgical group; Aldosterone: 710 pmol/L and ARR 127 in the medical group, *p* = 0.007 and *p* = 0.02) (Table 1). This supports the idea that adenomas secrete more aldosterone and could explain a higher proportion of cardiovascular complications compared to bilateral adrenal hyperplasia. However, in the subgroup of patients with unilateral primary hyperaldosteronism treated medically, the aldosterone level was also 765 pmol/L. The difference was not significant, likely due to the small sample size. This excess morbidity is resolved with adrenalectomy, highlighting the significance of differentiating PA subtypes in these patients. In our cohort, all the patients we operated on had unilateral PA, whereas 35 patients presented with suspected bilateral adrenal hyperplasia in the medical group, which may explain the lower prevalence of coronary heart disease in this group. Moreover, we observed that patients with a history of coronary disease had a longer progression of hypertension before being diagnosed with PA diagnosis compared to patients without coronary disease. Nevertheless, it is important to note that the date of the coronary events was not available. We should also note that overweight, a risk factor of CVE independent risk from PA, made no significant difference between the two groups. However, one of the limitations is that 29 patients in the medical group did not undergo an AVS.

Much of the research to date has examined the prevalence of CVE in patients with PA, comparing them with patients with essential hypertension [3, 4, 5]. Comparatively, little research has examined medical and surgical treatments on the occurrence of cardiovascular complications. The authors of a 2008 prospective study assessed long‐term cardiovascular outcomes after PA treatment in 54 patients at 7.4 years follow‐up [4]. They found no significant difference in the occurrence of a composite cardiovascular endpoint between patients treated surgically or medically (HR 1.26; 95% CI, 0.36–4.44; *p* = 0.71), which is consistent with our results. Similarly, Puar et al. [6] found in a retrospective analysis of 154 patients with unilateral PA that the incidence of CVE (with a composite score including acute myocardial infarction, coronary revascularization, stroke, atrial fibrillation, or congestive heart failure) was comparable between surgical (*n* = 86) and medical (*n* = 68) treatment, with the surgical group having an adjusted hazard ratio of 0.93 (95% CI: 0.32–2.67), *p* = 0.89. Muth et al. [7] reported similar cardiovascular and renal outcomes in patients who had received medical treatment or surgery.

However, other studies have presented evidence to the contrary. Wu et al. [8] conducted a retrospective study of all‐cause mortality in a large cohort of 3362 PA patients, with a mean follow‐up of 5.75 years. Four hundred and fifty‐two patients (13.4%) died, with an incidence rate of 23.4 per 1000 person‐years. Adrenalectomy patients had lower all‐cause mortality than medical patients (3.8% vs. 16.7%, *p *< 0.001). The surgical group also had a lower incidence of CVE (19.3% vs. 23.3%, *p *= 0.015). The authors also reported that medically treated patients were often under‐dosed and that a dose higher than 12.5 mg of spironolactone was required to reduce mortality in medically treated patients. More recently, a meta‐analysis [9] including 12 studies with a total of 6148 patients with PAH, evaluated the persistence of hypertension and the incidence of stroke (the composite of myocardial infarction, stroke, coronary revascularization, or hospitalization for heart failure) or all‐cause mortality between the two available treatments for PA. Of the 12 studies included, eight had hypertension control as their primary endpoint, while the other four had stroke or all‐cause mortality as their primary endpoint. The results showed a reduction in the number of strokes in patients with PA treated with adrenalectomy compared with those treated medically. The study did not find any significant difference in terms of CVE. Yet, it is important to note that the low number of events, the length of follow‐up, and the small number of patients have potentially obscured the beneficial outcomes of surgery. Furthermore, as mentioned above, patients treated surgically had a greater history of coronary heart disease, with fewer absolute strokes, despite equivalent cardiovascular risk factors.

We observed a significant reduction in blood pressure levels in the surgical group compared to the medical group. It is worth noting that various measurement methods, including office blood pressure, home blood pressure monitoring, and ambulatory blood pressure monitoring, were used. However, at inclusion, there was a significant lack of data for ABPM and HBPM values, mainly in the surgical group. Therefore, office BP was preferred for comparing the two groups at inclusion. During the last visit, and due to insufficient data, the patients were contacted by phone and were asked to complete a 3‐day HBPM. This data was collected, compared, and used in the key findings of this study. Sixteen patients could not be contacted. This disparity in the choice of measurement methods may constitute a bias, whereas the fact that all patients were asked to perform HBPM is a strength of this study. The number of antihypertensive classes in the surgical group was also significantly lower than in the medical group. Our findings are based on different treatments between the two groups, but we only reported the antialdosteronic treatment dose. Despite this limitation, our results are consistent with those in the literature. A 2012 study [2] compared 19 patients with operated unilateral lesions and 41 patients with medically managed bilateral lesions and found that surgery offered superior blood pressure and cardiac outcomes (left ventricular mass) after 2.5 years of follow‐up. Systolic and diastolic blood pressure decreased (*p *< 0.001) after both treatments, with absolute and percentage reductions significantly greater (*p* < 0.01) in the operated patients. Puar et al. [6] also found in a retrospective analysis of 154 patients with unilateral PA that both treatments (medical and surgical) improved BP and serum potassium levels control, but that surgery resulted in better BP control (systolic BP, 133 ± 11.7 vs. 137.9 ± 14.6 mmHg in the medical group, *p *= 0.02), and lower daily doses of antihypertensive drugs, 1.0 (IQR 0.0–2.0) versus 2.6 (IQR 0.8–4.3) in the medical group, *p *< 0.001. In addition, a systematic review [7] comparing the outcomes of PA patients after surgical versus medical treatment also showed a reduction in the use of antihypertensive drugs and an improvement in quality of life. In our study, surgery showed superiority in controlling hypokalemia. Initially, potassium levels were significantly lower in the surgical group.

In our study, surgical intervention demonstrated explicit effectiveness in controlling hypokalemia. Initially, the surgical group exhibited significantly lower potassium levels than the non‐surgical group. However, by the end of follow‐up, their potassium levels had increased significantly, exceeding those of the non‐surgical group, while the number of drugs and potassium supplementation were reduced. This data is consistent with other studies. For instance, in the primary aldosteronism surgical outcome (PASO) study [10], 84.2% of patients who underwent surgery achieved clinical success (cure or significant improvement in blood pressure control), with a biochemical success rate of 93.9%, corresponding to “correction of hypokalemia and normalization of the aldosterone‐renin ratio (ARR).”

One possible explanation for the effectiveness of surgical treatment could be that the blockade by antialdosterone treatment is not as extensive as surgery, which completely inhibits hypersecretion. Another hypothesis could be that mineralocorticoid receptors also have a high binding affinity for glucocorticoids. They would, therefore, be involved in the maturation of pre‐adipocytes into adipocytes [7]. This may be associated with a higher prevalence of hyperglycemia in PA patients compared with patients with essential hypertension [11]. However, there is no clear evidence that MRAs result in better glycemic and lipid control in PA patients than surgery. Recent studies [12] adrenalectomy in patients with PA has been shown to reverse osteoporosis, reduce the risk of metabolic syndrome, and improve quality of life. This could be attributed to reduced glucocorticoid levels after adrenalectomy and the correction of aldosterone excess. These observations confirm the importance of adrenalectomy in PA patients as the optimal approach to reducing cardiovascular risk. Similarly, surgical treatment of PA is safe, with low morbidity and a complication rate of between 2% and 10%. Antialdosteronic treatment can induce a further increase in aldosterone and trigger a vicious circle, leading to an insufficient effect of the medical treatment. This raises other issues, such as compliance with medication or the impossibility of prescribing adequate doses due to the frequent occurrence of dose‐dependent adverse effects (gynecomastia, dysmenorrhea, etc.). Patients who receive medical treatment for hypertension require more antihypertensive therapy, longer follow‐up periods, and more clinical visits to the specialist referral center than those treated surgically. However, they are more likely to receive better attention and benefit from comprehensive medical care, which includes the prevention of cardiovascular risk factors.

A key strength of the present study was to carefully report a relatively high cohort of patients followed for PA and to compare the occurrence of CVE according to the medical and surgical treatment. Only patients with proven PA were included, which avoids misdiagnosis of PA. In addition, all patients were contacted by phone, enabling CVE to be collected and confirmed, as well as the latest treatment and biological results. This helped to limit data collection errors and loss of data. However, we acknowledge that the patients may make errors depending on their understanding. Nevertheless, this was a valuable inclusion in the electronic medical records. Additionally, all patients were required to conduct a 3‐day HBPM, consisting of three morning and three evening measurements. This aimed to provide a more accurate blood pressure profile. However, we acknowledge that providing training for HBPM over the phone, without a subsequent in‑person verification of technique, may introduce variability and affect the reliability of the blood pressure readings. Nonetheless, this approach reflects real‑world clinical practice, where remote monitoring is increasingly used, and provides valuable information despite its constraints. The principal limitation of this paper lies in the number of cases used. Notwithstanding the relatively limited sample, the size of our cohort is consistent with most of the literature's studies, with the exclusion of two or three large cohorts [9]. Still, potential confounders may persist despite careful matching. Nevertheless, our findings remain consistent with existing literature. Additionally, it is important to note that our study is retrospective, and as such, is subject to the inherent shortcomings of such methodologies, which include missing data. It is important to highlight that the limited number of events, the duration of follow‐up, and the small sample size may have potentially masked the positive effects of surgery. Additionally, the study is limited in that we did not investigate treatment compliance or examine the long‐term measurement of plasma renin and aldosterone. A compliance questionnaire could have been used during the phone call. It is important to point out that our medical treatment group included both patients with unilateral and bilateral PA, whereas the surgical group comprised only patients with unilateral disease. This inevitably introduces a bias, as these forms of PA have distinct clinical profiles and prognoses. To address this, we analyzed a subgroup of patients with confirmed unilateral PA by AVS, although the small sample size limits the interpretation of the results. Nonetheless, this approach reflects clinical practice, where some patients presenting with a unilateral form due to a microadenoma may not be identified if AVS is not performed, especially when surgery is not an option. Moreover, unilateral and bilateral PA have different outcomes despite similar medical therapy. As a result, our findings apply primarily to patients with unilateral disease. In clinical reality, however, some patients with bilateral hyperplasia may have a microadenoma that remains undetected when catheterization is not performed. This underscores the importance of AVS to accurately determine the specific subtype of hyperaldosteronism

## 5 Conclusion

We demonstrated that surgery did not show a significant difference in the occurrence of cardiovascular events compared to medical treatment in patients with PA. However, there appeared to be a trend toward a reduction in the number of long‐term CVEs in patients who had more cardiovascular comorbidities at inclusion. Furthermore, surgery significantly improved blood pressure control with less medication and better control of serum potassium levels. To confirm these data, prospective studies would be necessary. Moreover it would be necessary to study the unilateral and bilateral forms of PA separately.

## Author Contributions

Sofia Benameur and Gabrielle Sarlon‐Bartoli contributed the central idea. Sofia Benameur wrote the initial draft of the paper. Sofia Benameur, Ngoc Anh Thu Nguyen, and Julien Bertolino analyzed the data. The remaining authors contributed to refining the ideas, carrying out additional analyses, and finalizing this paper. All authors gave final approval and agreed to be accountable for all aspects of the work, ensuring integrity and accuracy.

## Ethics Statement

The data collection was authorized by AP‐HM and was registered on the Health Data Access Portal under the number PADS22‐26, supervised by the National Commission for Information Technology and Civil Liberties.

## Consent

The author(s) collected consent from all included patients by telephone.

## Conflicts of Interest

The author(s) declared no potential conflicts of interest with respect to the research, authorship, and/or publication of this article.

## Supporting information



Appendix: Treatments.

## Data Availability

The authors have nothing to report.

## References

[1] G. A. Kline , J. L. Pasieka , A. Harvey , B. So , and V. C. Dias , “Medical or Surgical Therapy for Primary Aldosteronism: Post‐Treatment Follow‐Up as a Surrogate Measure of Comparative Outcomes,” Annals of Surgical Oncology 20, no. 7 (2013): 2274–2278, 10.1245/s10434-013-2871-3. 23504117

[2] G. Bernini , A. Bacca , V. Carli , et al., “Cardiovascular Changes in Patients With Primary Aldosteronism After Surgical or Medical Treatment,” Journal of Endocrinological Investigation 35, no. 3 (2012): 274–280, 10.3275/7611. 21422805

[3] P. Mulatero , S. Monticone , C. Bertello , et al., “Long‐Term Cardio‐ and Cerebrovascular Events in Patients With Primary Aldosteronism,” Journal of Clinical Endocrinology & Metabolism 98, no. 12 (2013): 4826–4833, 10.1210/jc.2013-2805. 24057288

[4] C. Catena , “Cardiovascular Outcomes in Patients With Primary Aldosteronism After Treatment,” Archives of Internal Medicine 168, no. 1 (2008): 80, 10.1001/archinternmed.2007.33. 18195199

[5] S. Savard , L. Amar , P. F. Plouin , and O. Steichen , “Cardiovascular Complications Associated With Primary Aldosteronism: A Controlled Cross‐Sectional Study,” Hypertension 62, no. 2 (2013): 331–336, 10.1161/HYPERTENSIONAHA.113.01060. 23753408

[6] T. H. Puar , L. M. Loh , W. J. Loh , et al., “Outcomes in Unilateral Primary Aldosteronism After Surgical or Medical Therapy,” Clinical Endocrinology 94, no. 2 (2021): 158–167, 10.1111/cen.14351. 33058182

[7] A. Muth , O. Ragnarsson , G. Johannsson , and B. Wängberg , “Systematic Review of Surgery and Outcomes in Patients With Primary Aldosteronism,” British Journal of Surgery 102, no. 4 (2015): 307–317, 10.1002/bjs.9744. 25605481

[8] V. C. Wu , S. M. Wang , C. H. Chang , et al., “Long Term Outcome of Aldosteronism After Target Treatments,” Scientific Reports 6 (2016): 32103, 10.1038/srep32103. 27586402 PMC5009379

[9] W. C. Huang , Y. Y. Chen , Y. H. Lin , and J. S. Chueh , “Composite Cardiovascular Outcomes in Patients With Primary Aldosteronism Undergoing Medical Versus Surgical Treatment: A Meta‐Analysis,” Frontiers in Endocrinology 12 (2021): 644260, accessed July 11, 2023, https://www.frontiersin.org/articles/10.3389/fendo.2021.644260. 34079522 10.3389/fendo.2021.644260 PMC8165438

[10] J. Burrello , A. Burrello , M. Stowasser , et al., “The Primary Aldosteronism Surgical Outcome Score for the Prediction of Clinical Outcomes After Adrenalectomy for Unilateral Primary Aldosteronism,” Annals of Surgery 272, no. 6 (2020): 1125, 10.1097/SLA.0000000000003200. 30672800

[11] F. Fallo , F. Veglio , C. Bertello , et al., “Prevalence and Characteristics of the Metabolic Syndrome in Primary Aldosteronism,” Journal of Clinical Endocrinology & Metabolism 91, no. 2 (2006): 454–459, 10.1210/jc.2005-1733. 16291704

[12] N. Sukor , C. Kogovsek , R. D. Gordon , D. Robson , and M. Stowasser , “Improved Quality of Life, Blood Pressure, and Biochemical Status Following Laparoscopic Adrenalectomy for Unilateral Primary Aldosteronism,” Journal of Clinical Endocrinology & Metabolism 95, no. 3 (2010): 1360–1364, 10.1210/jc.2009-1763. 20089615

