# Supplementary material for: Primary Aldosteronism and Long‐Term Cardiovascular Complications: Comparison of Medical Versus Surgical Treatment
Source: J Clin Hypertens (Greenwich). 2025 Aug 22;27(8):e70128. doi: 10.1111/jch.70128 (PMC12372980; doi:10.1111/jch.70128)
Supplement: Supplementary file 1 — Appendix: Treatments. [file JCH-27-e70128-s001.docx]

**Appendix : Treatments**

|  | | **Medical treatment** | **Surgical treatment** | **p-value** |
| --- | --- | --- | --- | --- |
| **Initial treatments** | Amiloride  Amiloride dosage | 0 (0%) | 3 (5,4%) | 0,24 |
|  |  | 0 (0%) | 6,66 ± 1.63 | 0,80 |
|  | Spironolactone  Spironolactone Dosage | 2 (4%) | 9 (16,4%) | 0,04 |
|  |  | 25 ± 4.95 | 52,7 ± 25.10 | 0,80 |
|  | Eplerenone  Eplerenone dosage | 1 (2%) | 0 (0%) | 0,48 |
|  |  | 75 ± 10.61 | 5± 0.67 | 0,32 |
|  | ACEi/ARB | 6 (11%) | 16 (29%) | 0,26 |
|  | Non-DHP CCBs | 9 | 1 | 0,02 |
|  | DHP CCBs | 41 | 43 | 0,47 |
|  | Thiazide diuretic | 2 | 5 | 0,44 |
|  | Alpha Blockers | 21 | 21 | 0,40 |
|  | Beta Blockers | 8 | 14 | 0,56 |
|  | Loop diuretic | 1 | 1 | 1 |
|  | Central antihypertensive | 11 | 11 | 0,80 |
| **Final treatments** | Amiloride  Amiloride dosage | 20 (42,5%) | 0 | 0 |
|  |  | 8,2 ± 4.9 | 0 | 4,06E-05 |
|  | Spironolactone  Spironolactone dosage | 18 (39,1%) | 4 (10,3%) | 0,002 |
|  |  | 38,5 ± 22 | 56,2 ± 23 | 0,04 |
|  | Eplerenone  Eplerenone dosage | 6 (13,0%) | 0 | 0,03 |
|  |  | 58,3± 21.6 | 0,00 | 0,03 |
|  | ACEi/ARB | 23 (48,9%) | 11 (28,2%) | 0,42 |
|  | Non-DHP CCBs | 1 (2,2%) | 2 (5,3%) | 0,43 |
|  | DHP CCBs | 30 (63,8%) | 13 (33,3%) | 0,007 |
|  | Thiazide diuretic | 12 (26,0%) | 3 (7,7%) | 0,07 |
|  | Alpha Blockers | 4 (8,7%) | 3 (7,7%) | 0,90 |
|  | Beta Blockers | 7 (15,2%) | 10 (25,6%) | 0,25 |
|  | Loop diuretic | 0 (0%) | 0 (0%) | 1 |
|  | Central antihypertensive | 1 (2,2%) | 2 (5,1%) | 0,59 |
|  | Number of treatments | 2,83 ± 1.8 | 1,23 ± 1.5 | 0,00005 |

ACEi : angiotensin-converting enzyme inhibitor; ARB : angiotensin receptor blocker; Non-DHP CCBs : Nondihydropyridine Calcium Channel Blockers ; DHP CCBs : DihydropyridineCalcium Channel Blockers
